# Dynamic multivariate patterns of brain structure–neuropsychiatric symptom associations in long COVID

**DOI:** 10.1093/braincomms/fcag232

**Published:** 2026-06-18

**Authors:** Wenrui Bao, Gengchen Ye, Tao Wang, Xingpu Quan, Qiange Zhu, Liming Fan, Haining Li, Wentao Zeng, Junya Mu, Ruiting Zhu, Jixin Liu, Yuchen Zhang, Xuan Niu

**Affiliations:** Department of Medical Imaging, the First Affiliated Hospital of Xi’an Jiaotong University, Xi’an, Shaanxi Province 710061, China; School of Future Technology, Xi’an Jiaotong University, Xi’an, Shaanxi Province 710049, China; Department of Medical Imaging, the First Affiliated Hospital of Xi’an Jiaotong University, Xi’an, Shaanxi Province 710061, China; Key Laboratory of Shaanxi Province for Craniofacial Precision Medicine Research, College of Stomatology, Xi’an Jiaotong University, Xi’an, Shaanxi Province 710004, China; Laboratory Center of Stomatology, College of Stomatology, Xi’an Jiaotong University, Xi’an, Shaanxi Province 710004, China; Department of Medical Imaging, College of Stomatology, Xi’an Jiaotong University, Xi'an, Shaanxi Province 710004, China; Department of Medical Imaging, the First Affiliated Hospital of Xi’an Jiaotong University, Xi’an, Shaanxi Province 710061, China; Department of Medical Imaging, the First Affiliated Hospital of Xi’an Jiaotong University, Xi’an, Shaanxi Province 710061, China; The Key Laboratory of Biomedical Information Engineering of Ministry of Education, Institute of Health and Rehabilitation Science, Xi’an Jiaotong University School of Life Science and Technology, Xi’an, Shaanxi Province 710049, China; PET/CT Center, the First Affiliated Hospital of Xi’an Jiaotong University, Xi’an, Shaanxi Province 710061, China; Department of Medical Imaging, the First Affiliated Hospital of Xi’an Jiaotong University, Xi’an, Shaanxi Province 710061, China; Department of Medical Imaging, the First Affiliated Hospital of Xi’an Jiaotong University, Xi’an, Shaanxi Province 710061, China; Department of Medical Imaging, the First Affiliated Hospital of Xi’an Jiaotong University, Xi’an, Shaanxi Province 710061, China; School of Future Technology, Xi’an Jiaotong University, Xi’an, Shaanxi Province 710049, China; School of Life Science and Technology, Xidian University, Xi'an Key Laboratory of Intelligent Sensing and Regulation of Trans-Scale Life Information, Xi’an, Shaanxi 710126, China; Department of Nuclear Medicine, the First Affiliated Hospital of Xi’an Jiaotong University, Xi’an, Shaanxi Province 710061, China; Department of Medical Imaging, the First Affiliated Hospital of Xi’an Jiaotong University, Xi’an, Shaanxi Province 710061, China

**Keywords:** long COVID, neuropsychiatric symptoms, cortical thickness, grey matter volume, regularized canonical correlation analysis

## Abstract

Long COVID presents with heterogeneous and persistent neuropsychiatric symptoms, suggesting possible shared neural underpinnings. However, the dynamic brain structure–symptom relationships and their potential for predicting long-term outcomes remain unclear. We conducted a longitudinal study of 144 individuals with long COVID (mean age: 37.8 ± 10.1 years, 48.6% male). All participants experienced mild COVID-19 and were not hospitalized. Structural MRI and comprehensive psychiatric and cognitive assessments were performed at 1, 2 and 12 months post-infection. A cohort of 68 healthy controls (mean age: 36.0 ± 10.3 years; 41.2% male) completed the same assessment protocol at baseline. Regularized canonical correlation analysis was employed to identify multivariate associations between 13 psychiatric and cognitive measures and both grey matter volume and cortical thickness, and to assess whether early structural features predicted symptom outcomes at 1 year. Neuropsychiatric symptoms were linked to coordinated structural covariance patterns across distributed brain regions in long COVID; these associations strengthened from 1 to 3 months post-infection and were absent in controls. A stable cognitive-affective symptom dimension showed the strongest brain–behaviour coupling. Notably, the neural substrates of this coupling diverged over time: grey matter volume associations remained localized to prefrontal-limbic circuits, whereas cortical thickness associations expanded to frontoparietal regions. Critically, reduced grey matter volume in the right cuneus and superior frontal gyri, along with decreased cortical thickness in the left supramarginal gyrus at 3 months post-infection, were significantly linked to poorer executive function and greater fatigue 1 year later. Our findings delineate evolving neuroanatomical signature of long COVID, where distinct patterns of grey matter volume and cortical thickness underpin a core symptom profile and predict long-term neuropsychiatric symptoms. These results provide insight into the neural mechanisms of long COVID and identify specific targets for monitoring and early intervention.

## Teaser

Bao *et al*. report that cognitive and psychiatric symptoms in long COVID are associated with time-varying patterns of brain structure. These brain–behaviour relationships are linked to 1-year outcomes, suggesting dynamic neural processes and potential targets for early identification

## Graphical Abstract

**Graphical Abstract:**
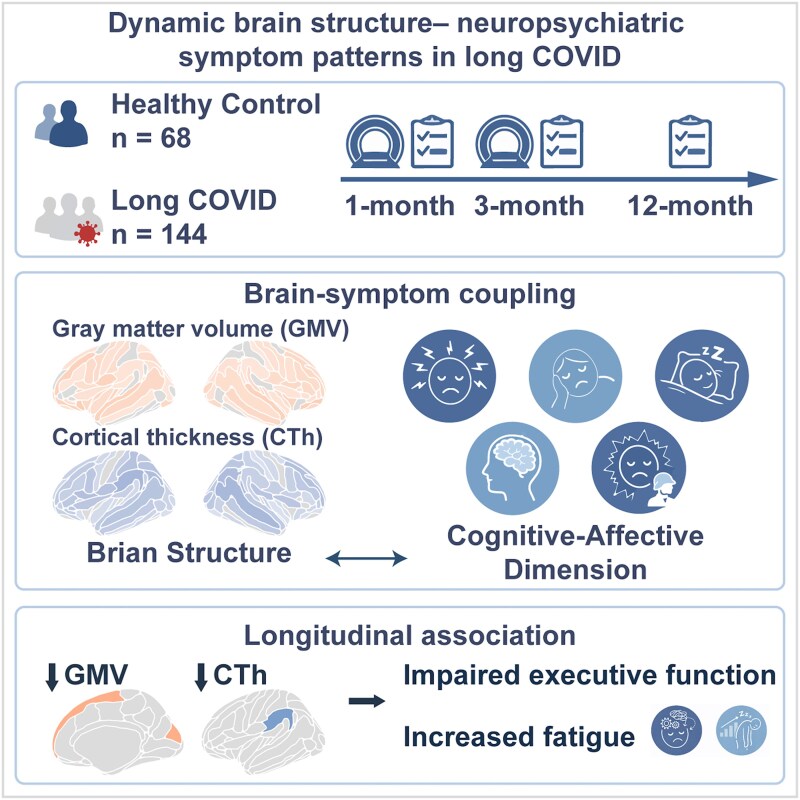

## Introduction

Neuropsychiatric manifestations are hallmark features of long COVID.^1,2^ Approximately 30% of individuals with mild COVID-19 experience persistent symptoms, including cognitive impairment, fatigue, sleep disturbances, anxiety, depression and post-traumatic stress disorder (PTSD), with <1% achieving complete recovery within 80 days.^3^ Cognitive impairment in long COVID is typically multidomain, featuring distinct subtypes with preferential deficits in attention, memory, or executive function.^4,5^ Fatigue, another prevalent and debilitating symptom, has been strongly linked to comorbid anxiety or depression.^6,7^ Beyond symptom severity, increasing evidence underscores fundamental differences in patients’ symptom perception.^8^ For instance, subjective fatigue complaints may be dissociated from objective performance-based measures related to attention and cognition. Notably, anosognosia (reduced awareness of deficits) has been proposed as a key contributor to the complex clinical phenotypes in long COVID.^9^ Collectively, heterogeneous symptom profiles, variable recovery trajectories and complex interactions between physiological and psychological factors may jointly contribute to the diverse presentations of long COVID.^10^ Despite growing epidemiological evidence on the clustering of long COVID symptoms, limited research on underlying mechanisms poses significant challenges to effective therapeutic strategies.^11^

Recent neuroimaging studies have revealed extensive brain structural alterations in long COVID patients.^12-14^ These changes may result from the persistence of severe acute respiratory syndrome coronavirus 2 (SARS-CoV-2) in various tissues and associated neuroinflammation, which disrupts glial and neuronal function, ultimately impairing neural circuits.^12,15,16^ The longitudinal MRI study from the UK Biobank reported significant reductions in cortical thickness (CTh) in olfactory and limbic regions in individuals with COVID-19, accompanied by executive function impairments 4 months post-infection.^17^ As a sensitive marker of local neuronal density and synaptic pruning, CTh exhibits region-specific variations that are closely linked to cognitive performance.^18^ Further studies have reported reduced CTh in the left superior temporal gyrus, a region involved in complex cognitive processing, in patients with neurological long COVID.^19^ Complementing CTh, grey matter volume (GMV) offers insights into more global structural changes related to development, atrophy, or inflammation.^20^ A hypothesis-driven region-of-interest (ROI) study found that reduced thalamic and basal ganglia volumes were related to core long COVID symptoms, including persistent fatigue, excessive daytime sleepiness and short-term memory deficits.^21^ Furthermore, a 3-month follow-up study identified additional GMV reductions in the left frontal, right temporal and left inferior parietal cortices among individuals with post-COVID-19 sleep disturbances.^22^

However, most existing studies focus on isolated neuropsychiatric symptoms, overlooking the overlapping and interconnected nature of long COVID symptomatology.^23,24^ Moreover, approaches relying on a single structural metric or a predefined ROI may fail to capture the distributed and coordinated nature of neural alterations in long COVID, potentially missing contributions from other brain regions within affected large-scale networks.^25^ These limitations highlight the need for a more comprehensive approach to understanding the neural mechanisms underlying long COVID. Although the 3-month post-infection period is critical for defining long COVID, persistent neuropsychiatric symptoms may correspond to relatively stable patterns of brain structural disruptions.^22^ However, the emergence and progression of these structural changes remain unclear. Investigating the dynamic relationship between brain structure and psychiatric and cognitive impairments from the earliest stages to the post-acute phase, even up to 1 year, could help identify key brain regions showing stable and persistent changes. These regions may serve as early, sensitive biomarkers for persistent long COVID symptoms. We hypothesize that complex neuropsychiatric symptoms in long COVID may share a common neural mechanism, and the multivariate patterns of brain structure-neuropsychiatric symptom associations will dynamically evolve from the acute phase to 3 months post-infection. Therefore, a longitudinal design integrating multidimensional symptom clusters and combined analyses of cortical thickness and grey matter volume may provide a more comprehensive understanding of brain alterations associated with long COVID.

Regularized Canonical Correlation Analysis (RCCA), a multivariate data-driven method, has been widely used to capture the underlying relationship between brain and behaviour.^25,26^ Importantly, regularization and integration with machine learning approaches have reduced overfitting and improved the generalizability and stability of RCCA. In this study, we aimed to identify multivariate associations between neuropsychiatric symptoms—such as cognitive impairments in executive function, working memory and episodic memory, as well as fatigue, sleep disturbances, anxiety, depression and PTSD—and brain structural metrics, including CTh and GMV, in individuals with long COVID at 1 and 3 months post-infection and in uninfected healthy controls. Moreover, we investigated whether early structural markers in key brain regions demonstrated behavioural relevance 1 year post-infection, providing insights into their long-term implications.

## Materials and methods

### Study design and subject recruitment

This prospective study was conducted across nine hospitals in China from January 2023 to January 2024. The study protocol was approved by the Ethics Committee of the First Affiliated Hospital of Xi'an Jiaotong University (No. XJTU1AF2023LSK-013), and all participants provided voluntary written informed consent.^27^

A total of 144 individuals meeting the World Health Organization (WHO) definition of long COVID were enrolled. Long COVID was defined as the presence of at least 1 symptom persisting for ≥12 weeks after confirmed or probable SARS-CoV-2 infection without an alternative explanation.^28^ Symptomatology was assessed using the Long COVID Symptom Severity Scale (LC-SSS), a validated tool developed by our team covers 44 symptoms across six domains: Cardiopulmonary, Psychological, Neurological, Gastrointestinal, Pain and Ear-Nose-Throat (ENT).^29^ Participants were classified as having long COVID if their LC-SSS score was >0,^30^ with fatigue, cognitive disturbances and sleep problems being the most frequently reported symptoms (Supplementary Fig. 1). This long COVID cohort was derived from a larger longitudinal study of patients with mild SARS-CoV-2 infection confirmed by RT-PCR or serology. For the present analysis, data from the 1- and 3-month follow ups, including MRI and neuropsychiatric assessments, were included. At 1 year post-infection, 54 participants completed the follow-up neuropsychiatric assessment.

A contemporaneous control group of 68 healthy individuals, confirmed as repeatedly RT-PCR negative with no history of COVID-19, was recruited and matched to the patient group by age, sex and education. Controls underwent the same neuropsychiatric evaluations and MRI scans, but only at baseline. Exclusion criteria for all participants included age ≤18 or ≥65 years, history of neurological or psychiatric disorders, chronic pulmonary or central nervous system diseases, left-handedness and MRI contraindications (e.g. metal implants, pacemakers, claustrophobia, or pregnancy). To ensure close temporal alignment between symptom evaluation and imaging, neuropsychiatric assessments and MRI scans were performed within 48 h for both groups.

### Comprehensive assessment batteries for neuropsychiatric symptoms

To comprehensively evaluate psychiatric symptoms, we performed the following standardized scales: (1) the Multidimensional Fatigue Inventory-20 (MFI-20) to measure fatigue severity;^31^ (2) the Impact of Events Scale-Revised (IES-R) for PTSD symptoms;^32^ (3) the Beck Depression Inventory (BDI) and the Beck Anxiety Inventory (BAI) to evaluate depression and anxiety severity, respectively;^33,34^ and (4) the Pittsburgh Sleep Quality Index (PSQI) for sleep disturbances;^35^

Cognitive function was assessed using objective tests: (5) the Forward and Backward Digit Span (FDS/BDS) for short-term and verbal working memory;^36^ (6) the Auditory Verbal Learning Test-Hua Shan version (AVLT-H) for immediate recall, short-term and long-term delayed recall and recognition^37^; (7) the Trail-Making Test Part A (TMT-A) and Part B (TMT-B) to evaluate cognitive processing speed and executive function.^38^

### MRI acquisition and preprocessing

MRI data were acquired on a 3.0T scanner using a high-resolution T1-weighted sequence, with imaging parameters detailed in Supplementary Table 2. To reduce non-biological variability arising from differences in scanners and imaging protocols across multiple centres, datasets were harmonized using the ComBat technique.^39^ Structural MRI preprocessing was performed in FreeSurfer software (v7.4, https://surfer.nmr.mgh.harvard.edu), including motion correction, skull stripping, automated Talairach transformation and intensity normalization. This pipeline facilitated the identification and labeling of cortical and subcortical structures. Cortical thickness and grey matter volume were extracted using the Destrieux atlas.^40^

### Regularized canonical correlation analysis

**Figure 1:**
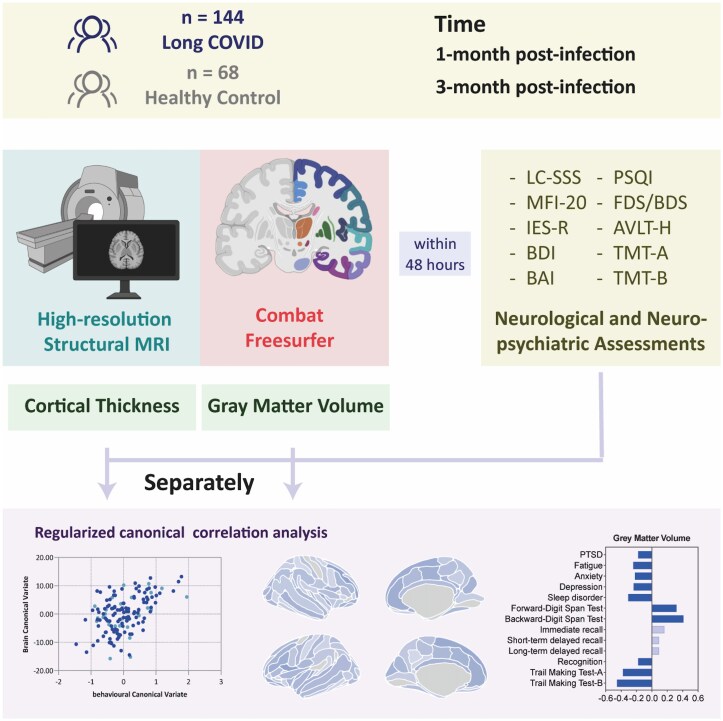
**Study design and regularized canonical correlation analysis (RCCA) framework for investigating the relationships between brain structure and neuropsychiatric symptoms in individuals with long COVID and healthy controls.** LC-SSS, Long COVID Symptom Severity Scale; PSQI, Pittsburgh Sleep Quality Index; MFI, Multidimensional Fatigue Inventory; FDS/BDS, Forward and Backward Digit Span; IES-R, The Impact of Event Scale—Revised; AVLT-H, Auditory Verbal Learning Test-Hua Shan version; BDI, Beck Depression Inventory; BAI, Beck Anxiety Inventory; TMT-A and TMT-B, Trail-Making Test Part A and Part B.

Regularized canonical correlation analysis (RCCA) was employed to investigate the multidimensional relationships between neuropsychiatric symptoms and brain structure. Thirteen neuropsychiatric variables—eight cognitive (Digit Span Forward and Backward total scores, Immediate, Short-delay, and Long-delay recall scores, Recognition score, TMT-A and TMT-B scores) and five psychiatric measures (IES-R, MFI, BAI, BDI and PSQI total scores)—were analysed in relation to CTh and GMV across 148 cortical regions. In the RCCA framework, two data matrices were modelled: *X* (13 neuropsychiatric variables × *n* participants) and *Y* (148 cortical regions × *n* participants, representing CTh/GMV features). The analysis identified two canonical variates—a behavioural variate derived from X(u) and a brain variate derived from Y(v)—whose linear combinations were optimized to maximize their correlation. Analyses were performed separately for long COVID participants and healthy controls (Fig. 1).

RCCA incorporates L2-norm regularization, similar to ridge regression, to penalize the sum of squared weights. Unlike methods that shrink weights to zero, RCCA encourages smaller weights, effectively reducing multicollinearity.^25^ The optimization problem for our RCCA model is formulated as follows:


$$\underset{w_{x},w_{y}}{max}\;\frac{corr(Xw_{x},Yw_{y})\sqrt{var(Xw_{x})}\sqrt{var(Yw_{y})}}{\sqrt{(1-c_{x})var(Xw_{x})+c_{x}}\sqrt{(1-c_{y})var(Yw_{y})+c_{y}}}$$

where $c_{x}$ and $c_{y}$ are hyperparameters controlling the level of regularization. When $c_{x}=c_{y}=0$, the model behaves as traditional Canonical Correlation Analysis (CCA), whereas $c_{x}=c_{y}=1$ enforces full regularization akin to Partial Least Squares (PLS). This framework enables a continuous transition between CCA and PLS, balancing model flexibility and stability for robust bimodal data integration.

To determine the optimal regularization parameters $\left(c_{x},c_{y}\right)$, a nested 5 × 5 cross-validation framework was implemented. The outer loop divided the dataset into five folds, using 80% of the data as an optimization set and the remaining 20% as a hold-out test set for evaluating model generalization. Within each optimization set, an inner 5-fold cross-validation identified the combination of $c_{x}\;$ and $c_{y}$ that maximized the mean canonical correlation across validation folds. For each outer fold, the RCCA model with optimal hyperparameters was retrained on the entire optimization set and evaluated on the hold-out test set.

Permutation testing with 1 000 iterations was conducted to assess the statistical significance of the latent correlation. During testing, rows of the behavioural and imaging data were shuffled independently to generate null distributions of the canonical correlations. The RCCA module used in this study was implemented from CCA/PLS toolkit developed by the Machine Learning & Neuroimaging Laboratory, Centre for Medical Imaging Computing, Department of Computer Science, University College London (https://github.com/anaston/cca_pls_toolkit).

### Interpretation of canonical variables

Under L2 regularization, weight coefficients $\left(w_{x},w_{y}\right)$ may become unstable in the presence of multicollinearity among variables. In contrast, canonical loadings $(L_{X}=corr(X,u);L_{y}=corr(Y,v));$ and cross-loadings $(CL_{X}=corr(X,v);CL_{y}=corr(Y,u));$ were computed to quantify the contribution of each original variable to within-domain and cross-domain canonical variates, providing more robust and interpretable indices of association strength between observed variables and the latent canonical dimensions. These correlations highlight the cortical regions and neuropsychiatric measures most strongly associated with the brain–behaviour relationships captured by the RCCA model.

To facilitate visualization of the most prominent brain–behaviour associations, we highlighted the top 5% of canonical cross-loadings (absolute magnitude) for CTh and GMV across the 148 cortical regions. This display-based threshold was used to illustrate the strongest regional contributors to the brain–behaviour pattern. Detailed regional cross-loadings and corresponding *P* values are provided in Supplementary Tables 5 and 6.

### Statistical analysis

The normality of continuous variables was assessed using the Kolmogorov–Smirnov test. Normally distributed variables are presented as mean ± SD, non-normally distributed variables as median (25th–75th interquartile range), and categorical variables as number (%). Group differences between long COVID patients and healthy controls were assessed using independent samples t-tests for normally distributed variables and Mann–Whitney U tests for non-normally distributed variables, while categorical variables were compared using Chi-square (χ^2^) or Fisher’s exact tests, as appropriate.

To examine whether key brain structural features at 3 months predicted long-term neuropsychiatric symptoms, we conducted partial correlation analyses between the top 5% of cross-loading regions for GMV and CTh and neuropsychiatric symptoms assessed at 1 year. Analyses were controlled for sex, age, years of education, estimated total intracranial volume (eTIV), and the mean CTh of both hemispheres at 3 months.

For these partial correlation analyses, the Benjamini-Hochberg false discovery rate (FDR) procedure was applied to control for multiple comparisons across brain regions and symptom variables. The FDR method was chosen over more conservative corrections (e.g. Bonferroni) to balance Type I errors and statistical power in exploratory neuroimaging analyses. Only correlations survived FDR correction (*P* < 0.05) are discussed in the main text as statistically significant predictors.

## Results

### Group differences in demographic characteristics, neuropsychiatric assessments, and brain morphometry in long COVID participants and healthy controls

A total of 144 long COVID participants (mean age: 37.8 ± 10.1 years; 48.6% male) and 68 non-infected healthy controls (HCs; mean age: 36.0 ± 10.3 years; 41.2% male) were included. Demographic characteristics, neuropsychiatric assessments and morphological brain measures are summarized in Table 1. Compared with HCs, long COVID participants exhibited significantly higher scores for event impact, fatigue, sleep disturbances and anxiety at 3 months post-infection (all FDR-corrected *P* < 0.05). At the 1-year follow-up, most symptoms had alleviated although sleep disturbances remained highly prevalent. In terms of cognitive function, long COVID participants demonstrated a persistent decline in recognition function from the 3-month assessment to the 1-year follow-up. No significant differences were observed in morphological brain measures, including eTIV, left hemisphere mean thickness, and right hemisphere mean thickness, between long COVID individuals at 1 and 3 months post-infection and HCs (Table 1 and Supplementary Table 1).

**Table 1:** Comparison of demographic characteristics, neuropsychiatric assessments and brain morphometric measures between long COVID participants (at 1 month, 3 months and 1-year post-infection) and healthy controls (baseline)

Characteristic	HCs (*n* = 68)	1 month (*n* = 144)	*P* Value	3 months (*n* = 144)	*P* Value	1 year (*n* = 54)	*P* Value
Demographic							
Age, y	35.99 ± 10.28	37.79 ± 10.11	0.208	37.87 ± 10.06	0.208	38.81 ± 8.85	0.114
Female, *n* (%)	40 (58.8%)	74 (51.4%)	0.193	74 (51.4%)	0.193	24 (45.3%)	0.139
Education, y	16.09 ± 2.66	16.47 ± 3.11	0.385	16.47 ± 3.11	0.385	16.21 ± 2.53	0.803
IES-R	9.63 ± 11.88	12.35 ± 14.00	0.168	14.62 ± 13.95	**0**.012**	12.83 ± 14.67	0.188
MFI	40.03 ± 12.80	51.83 ± 14.44	<0.001**	46.29 ± 13.97	**0**.002**	43.42 ± 14.44	0.175
PSQI	5.08 ± 2.99	7.14 ± 3.83	<0.001**	7.02 ± 3.77	<0.001**	6.43 ± 3.92	**0**.033*
BAI	2.90 ± 3.33	5.75 ± 6.01	<0.001**	4.10 ± 4.60	**0**.034*	3.02 ± 4.70	0.866
BDI	5.31 ± 6.20	7.98 ± 7.74	**0**.014*	5.54 ± 6.76	0.810	4.49 ± 6.25	0.474
Forward Digit Span	8.47 ± 1.52	8.55 ± 1.45	0.720	8.60 ± 1.44	0.557	8.71 ± 1.81	0.428
Backward Digit Span	5.97 ± 1.65	5.83 ± 1.68	0.558	6.02 ± 1.79	0.845	6.21 ± 1.89	0.456
Trail Making Test A (s)	39.50 ± 14.25	42.71 ± 20.09	0.237	40.26 ± 16.53	0.743	45.66 ± 22.88	0.072
Trail Making Test B (s)	98.09 ± 56.27	92.90 ± 46.46	0.479	90.28 ± 47.38	0.293	92.28 ± 33.70	0.508
AVLT-H							
Immediate recall	25.15 ± 6.00	25.08 ± 6.14	0.937	27.37 ± 5.73	**0**.010**	26.21 ± 5.67	0.324
Short delayed recall	9.34 ± 2.11	9.12 ± 2.37	0.514	9.64 ± 2.03	0.321	9.09 ± 2.16	0.537
Long delayed recall	9.21 ± 2.47	9.14 ± 2.31	0.847	9.56 ± 2.15	0.284	8.73 ± 2.46	0.294
Recognition	22.94 ± 2.38	21.85 ± 2.97	**0**.009*	20.85 ± 5.48	**0**.003**	17.15 ± 7.56	<0.001**

BMI, body mass index; IES-R, The Impact of Event Scale—Revised; MFI, Multidimensional Fatigue Inventory; PSQI, Pittsburgh Sleep Quality Index; BAI, Beck Anxiety Inventory; BDI, Beck Depression Inventory; AVLT-H, Auditory Verbal Learning Test-Hua Shan version; independent-sample t-tests were conducted to compare long COVID participants at each follow-up (1 month, 3 months and 1 year post-infection) with baseline healthy controls. Reported *P*-values correspond to these comparisons. Statistically significant results are highlighted in bold. *P < 0.05 (uncorrected); **FDR-corrected *P* < 0.05.

### Multivariate associations between neuropsychiatric symptoms and grey matter volume at 1 month post-infection in individuals with long COVID and HCs

No significant multivariate associations were observed between neuropsychiatric symptoms and GMV in HCs, with a mean out-of-sample canonical correlation of −0.184 ± 0.336 across folds (all *P* > 0.05). In contrast, long COVID participants showed distinct associations between symptom severity and GMV.

**Figure 2:**
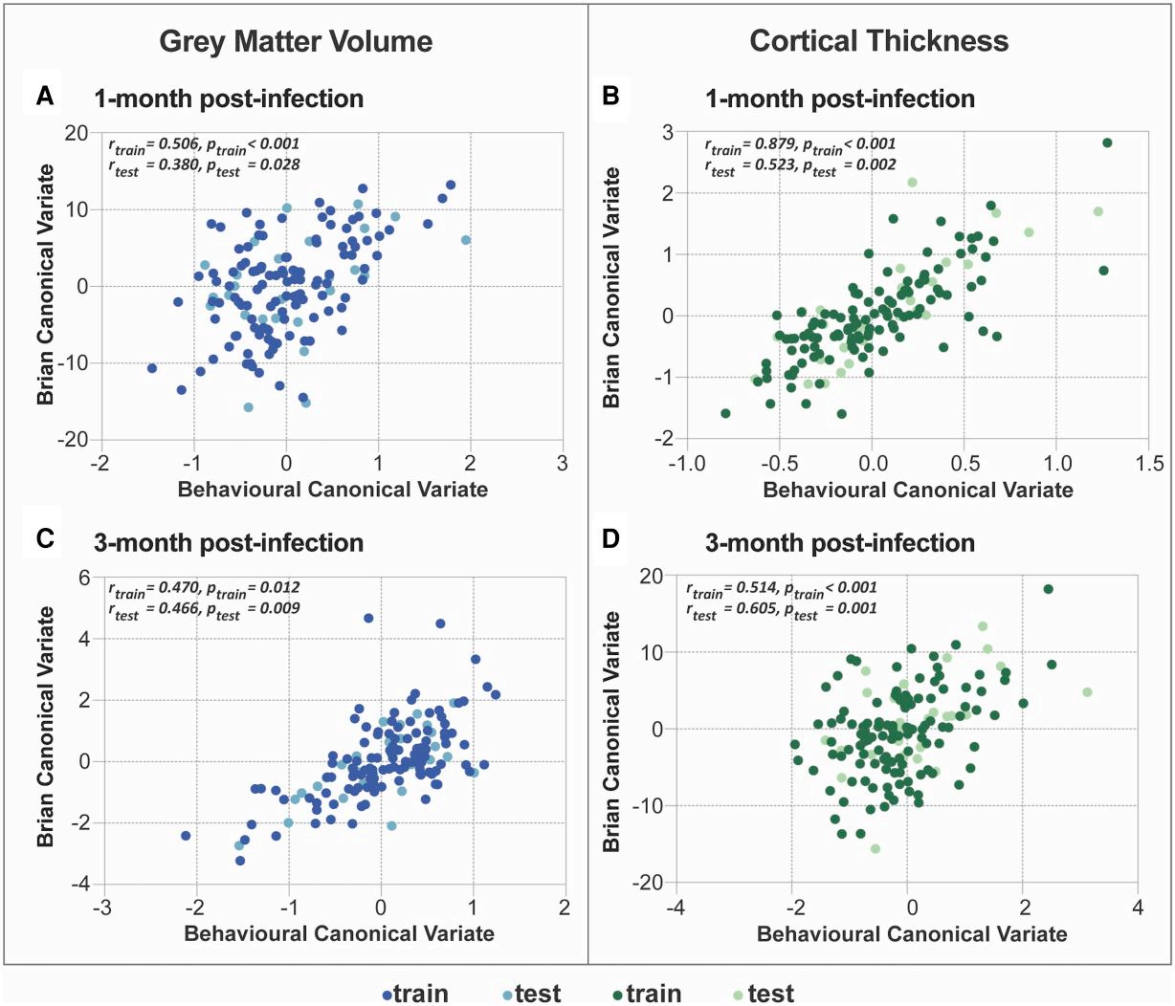
**Latent dimensions of neuropsychiatric symptoms and brain structure in individuals with long COVID (*n* = 144).** (**A**) and (**C**) show associations between neuropsychiatric symptoms and grey matter volume (GMV) at 1 and 3 months post-infection, respectively. (**B**) and (**D**) Show associations between neuropsychiatric symptoms and cortical thickness (CTh) at the corresponding time points. Each scatterplot depicts individual participants’ brain and behavioural canonical variate scores derived from the regularized canonical correlation analysis (RCCA) model. Dark-coloured points represent the training set, and light-coloured points represent the test set in the cross-validation framework. Statistical significance was evaluated using permutation testing (1000 permutations). The canonical correlation coefficients (r) and corresponding *P* values for each panel are as follows: **A** (train: r = 0.506, *P* < 0.001; test: r = 0.380, *P* = 0.028), **B** (train: r = 0.879, *P* < 0.001; test: r = 0.523, *P* = 0.002), **C** (train: r = 0.470, *P* = 0.012; test: r = 0.466, *P* = 0.009) and **D** (train: r = 0.514, *P* < 0.001; test: r = 0.605, *P* = 0.001). Results are visualized for illustrative purposes.

**Figure 3:**
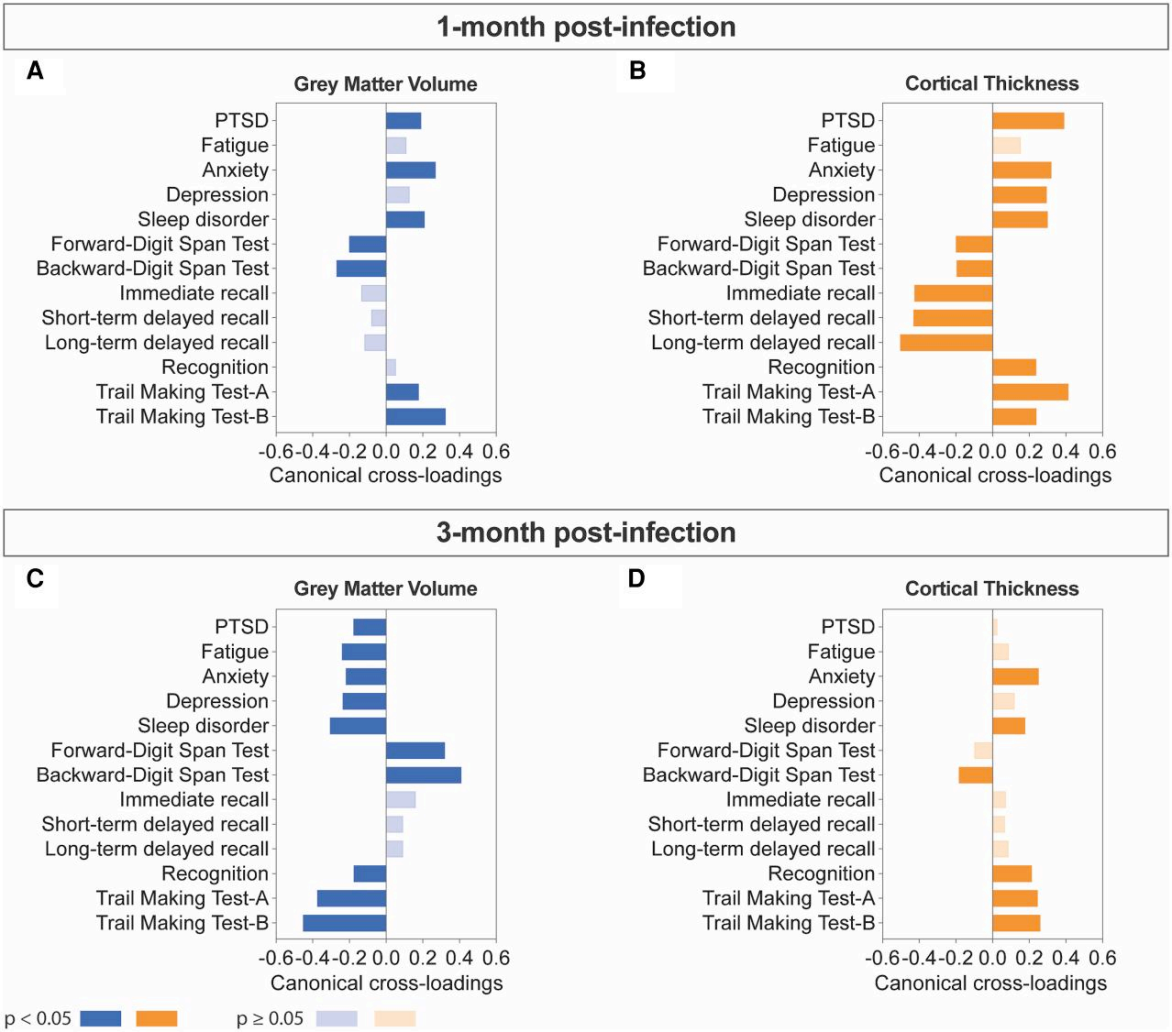
**Canonical cross-loadings of neuropsychiatric symptoms in individuals with long COVID (*n* = 144).** Bar plots illustrate the canonical cross-loadings of individual neuropsychiatric symptoms on the primary brain canonical variate. (**A**) and (**C**) show results for grey matter volume (GMV) at 1 month and 3 months post-infection, respectively, whereas (**B**) and (**D**) show results for cortical thickness (CTh) at the corresponding time points. Bar length reflects the magnitude of the cross-loading for each symptom, and colour intensity indicates the corresponding permutation-based *P* value, with darker colours representing stronger statistical significance. Statistically significant loadings (*P* < 0.05) are highlighted. Cross-loadings were derived from the regularized canonical correlation analysis (RCCA) model, and statistical significance was assessed using permutation testing (1000 iterations). The detailed canonical cross-loadings and corresponding *P* values for each neuropsychiatric symptom are provided in Supplementary Table 7.

At 1-month post-infection in long COVID participants, the mean out-of-sample canonical correlation was 0.139 ± 0.216, with 1/5 folds significant at *P* < 0.05 (Supplementary Table 3). The best performing fold showed a canonical correlation of r = 0.380 and a corresponding *P*-value of 0.028 (Fig. 2A). Examination of canonical loadings and cross-loadings indicated that several behavioural variables, including executive function (TMT-B, CLx = 0.325), working memory (BDS and FDS, CLx = −0.270 and −0.201 respectively) and psychiatric symptoms (anxiety and sleep disturbances, CLx = 0.269 and 0.210 respectively), exhibited both high canonical loadings (Supplementary Fig. 2A) and high canonical cross-loadings (Fig. 3A). These findings suggest that this symptoms cluster may represent a key behavioural contributor linking neuropsychiatric symptom severity with underlying structural brain covariance. Detailed canonical loadings and cross-loadings with corresponding *P* values are provided in Supplementary Tables 7 and 8.

**Figure 4:**
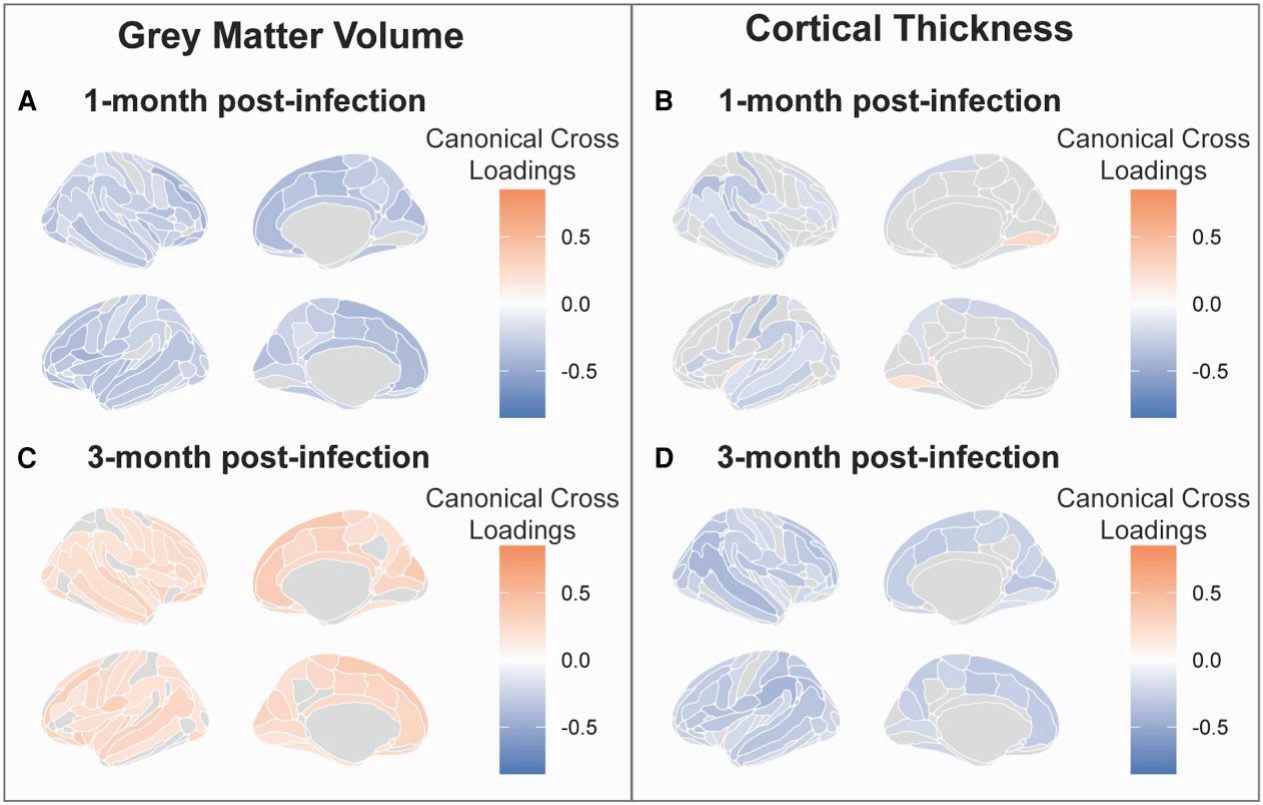
**Canonical cross-loadings of brain regions in individuals with long COVID (*n* = 144).** Brain maps display regional canonical cross-loadings associated with the primary brain–behaviour canonical variate. (**A**) and (**C**) show grey matter volume (GMV)–behaviour associations at 1 and 3 months post-infection, respectively. (**B**) and (**D**) show cortical thickness (CTh)–behaviour associations at the corresponding time points. Colour intensity reflects the magnitude and direction of the cross-loadings (warm colours indicate positive associations; cool colours indicate negative associations). Statistical significance of the canonical correlations was assessed using permutation testing (1000 permutations). Results are shown for the optimal model derived from the cross-validation framework. The detailed regional canonical cross-loadings, thresholded at the top 5% and 10% of the distribution, along with their corresponding *P* values, are provided in Supplementary Tables 5 and 6.

The top 5% of cross-loadings predominantly localized to regions within the prefrontal-limbic circuitry and primary sensory areas: the left inferior frontal gyrus, opercular part (CLx = −0.447), the right middle frontal gyrus (CLx = −0.441), the left superior frontal gyrus (CLx = −0.403), the left transverse temporal gyrus (Heschl's gyrus) (CLx = −0.390), the right superior frontal gyrus (CLx = −0.390), the left anterior cingulate gyrus and sulcus (CLx = −0.388), the right cuneus (CLx = −0.387) and the right anterior cingulate gyrus and sulcus (CLx = −0.386) (Fig. 4A). Notably, the superior frontal gyrus, middle frontal gyrus and anterior cingulate cortex were also among the top 5% of canonical loadings (Supplementary Fig. 3A), suggesting strong contributions to both brain and behavioural domains.

### Multivariate associations between neuropsychiatric symptoms and grey matter volume at 3 months post-infection in individuals with long COVID

The multivariate brain–behaviour relationship strengthened from 1 to 3 months post-infection. At 3 months post-infection, the mean out-of-sample canonical correlation was 0.316 ± 0.104, with 2/5 folds significant at *P* < 0.05 (Supplementary Table 3). The best performing fold showed a canonical correlation of r = 0.466 and a corresponding *P*-value of 0.009 (Fig. 2C). Examination of canonical loadings and cross-loadings indicated that the same core behavioural cluster—encompassing executive dysfunction (TMT-B and TMT-A, CLx = −0.453 and −0.375, respectively), working memory deficits (BDS and FDS, CLx = 0.410 and 0.320, respectively), PSQI and fatigue (CLx = −0.306 and −0.240, respectively)—continued to show the highest cross-loadings, indicating the stability of this neurobehavioural cluster (Fig. 3C; Supplementary Tables 7 and 8). However, the direction of their association with the neural variate reversed compared with 1 month post-infection.

The spatial distribution of the top 5% of cross-loadings remained largely consistent with that observed at 1 month, predominantly localizing to regions within the prefrontal-limbic circuitry and primary sensory areas, however, the direction of the associations was reversed: the right superior frontal gyrus (CLx = 0.392), the right cuneus (CLx = 0.376), the left superior frontal gyrus (CLx = 0.366), the right anterior cingulate gyrus and sulcus (CLx = 0.356), the right orbital gyrus (CLx = 0.345), the left anterior circular sulcus of the insula (CLx = 0.342), the left middle frontal gyrus (CLx = 0.331) and the right lateral superior temporal gyrus (CLx = 0.321) (Fig. 4C). Notably, the superior frontal gyrus, middle frontal gyrus, anterior cingulate cortex and cuneus were also among the top 5% of canonical loadings (Supplemental Fig. 3C), underscoring their consistent involvement across both post–COVID-19 stages.

### Multivariate associations between neuropsychiatric symptoms and cortical thickness at 1 month post-infection in individuals with long COVID and HCs

Consistent with the GMV results, no significant multivariate associations were observed between neuropsychiatric symptoms and cortical thickness in HCs, with a mean out-of-sample canonical correlation of 0.045 ± 0.291 across folds (all *P* > 0.05). In contrast, long COVID participants showed distinct associations between symptom severity and CTh.

At 1-month post-infection, the mean out-of-sample canonical correlation was 0.413 ± 0.099, with 4/5 folds reaching statistical significance (*P* < 0.05) (Supplementary Table 4). The best-performing fold yielded a canonical correlation of r = 0.523 (*P* = 0.002) (Fig. 2B). Examination of both canonical loadings and cross-loadings revealed that the dominant contributors were primarily cognitive variables, including episodic memory measures—long-term delayed recall, short-term delayed recall and immediate recall (CLx = −0.503, −0.431 and −0.426, respectively)—and executive function (TMT-A, CLx = 0.413). Psychiatric symptoms, particularly post-traumatic stress (PTSD, CLx = 0.390) and anxiety (CLx = 0.320), also exhibited high canonical loadings and cross-loadings, underscoring their contribution to the observed multivariate pattern (Fig. 3B and Supplemental Fig. 2B; Supplementary Tables 7 and 8).

The top 5% of cross-loadings showed a widespread spatial distribution across the frontal, parietal and temporal cortices: the left postcentral gyrus (CLx = −0.364), the right lateral superior temporal gyrus (CLx = −0.357), the right angular gyrus (CLx = −0.354), the right postcentral gyrus (CLx = −0.345), the left precentral gyrus (CLx = −0.322), the right supramarginal gyrus (CLx = −0.303), the right planum temporale (CLx = −0.296) and the left supramarginal gyrus (CLx = −0.286) (Fig. 4B). Notably, the precentral gyrus, postcentral gyrus and subregions of the inferior parietal lobule—including the angular and supramarginal gyri—were also among the top 5% of canonical loadings (Supplemental Fig. 3B).

### Multivariate associations between neuropsychiatric symptoms and cortical thickness at 3 months post-infection in individuals with long COVID

Compared with the 1-month results, the overall multivariate brain-behaviour relationship weakened at 3 months post-infection, with a mean out-of-sample canonical correlation of 0.294 ± 0.218 and 3/5 folds reaching statistical significance (*P* < 0.05) (Supplementary Table 4). However, the best-performing fold showed a higher canonical correlation of r = 0.604 (*P* = 0.001) (Fig. 2D). Examination of canonical loadings and cross-loadings indicated that cognitive variables remained the predominant contributors—particularly executive dysfunction (TMT-B and TMT-A, CLx = 0.260 and 0.245 respectively), episodic memory and working memory deficits (recognition and BDS, CLx = 0.215 and −0.184). In addition, anxiety (CLx = 0.252) showed an increased contribution compared with the 1-month results, alongside sleep disorder (CLx = 0.178) (Fig. 3D; Supplementary Tables 7 and 8).

The top 5% of cross-loadings exhibited a spatial shift from the sensorimotor network towards higher-order cognitive and affective regulatory areas from 1 to 3 months post-infection. However, the inferior parietal lobule—particularly the supramarginal and angular gyri—remained consistently within the top 5% across both timepoints: the left supramarginal gyrus (CLx = −0.415), the right superior temporal sulcus (CLx = −0.403), the right angular gyrus (CLx = −0.381), the left angular gyrus (CLx = −0.375), the right middle frontal gyrus (CLx = −0.358), the left inferior frontal gyrus, pars opercularis (CLx = −0.353), the right superior occipital gyrus (CLx = −0.346) and the left postcentral sulcus (CLx = −0.346) (Fig. 4D). Notably, the left supramarginal gyrus also ranked among the top 5% of canonical loadings (Supplemental Fig. 3D), underscoring its stable contribution to the brain–behaviour association pattern.

### Partial correlations between 3-month top 5% cross-loading regions and 1-year behavioural measures in individuals with long COVID

Controlling for sex, age, years of education, eTIV and mean cortical thickness of both hemispheres, we identified several significant associations between brain structural features at 3 months and behavioural outcomes at 1 year. Specifically, the GMV of the right cuneus was negatively correlated with executive function (TMT-B; r = −0.406, FDR-corrected *P* = 0.016). The GMV of the right superior frontal gyrus was negatively correlated with fatigue (MFI; r = −0.362, FDR-corrected *P* = 0.040). In addition, the CTh of the left inferior parietal–supramarginal gyrus was negatively correlated with executive function (TMT-B; r = −0.333, FDR-corrected *P* = 0.038).

## Discussion

In this longitudinal study, we found that (1) Neuropsychiatric symptoms of long COVID were associated with coordinated patterns of brain structural covariance across multiple regions, and these associations appeared to strengthen from 1 to 3 months after infection. No such associations were observed in non-infected HCs. (2) A cognitive disorder–affective symptom cluster contributed most prominently to these associations. Spatially, GMV-related covariance patterns were primarily identified within prefrontal–limbic circuits, whereas cortical thickness–related patterns progressively extended to fronto-parietal areas. (3) Structural variations in key brain regions during the early post-COVID phase showed longitudinal associations with later neuropsychiatric outcomes. Specifically, reduced GMV in the right cuneus and superior frontal gyri, along with decreased CTh in the left supramarginal gyrus at 3 months, were linked to poorer executive function and greater fatigue at the 1-year follow-up.

A substantial proportion of individuals experience long COVID, a post-acute syndrome characterized by persistent neuropsychiatric symptoms.^1^ Consistent with other epidemiological studies, participants with long COVID in our cohort exhibited more severe PTSD, fatigue, sleep disturbances and anxiety, as well as poorer recognition performance, compared with non-infected HCs. Importantly, these individuals had already shown marked fatigue, sleep disturbances, anxiety and depressive symptoms as early as the acute phase (1 month after infection). This pattern suggests that acute-phase symptom severity may serve as an early clinical indicator of long COVID risk, aligning with reports that greater symptom burden during the acute phase increases the likelihood of developing long COVID.^41^ Such early symptom burden may partly reflect acute inflammatory responses to infection, as elevated TNF-α levels during the acute phase have been linked to the subsequent emergence of cognitive impairment several months following COVID-19.^42^

When exploring the neural mechanisms of long COVID, we found no significant multivariate associations between neuropsychiatric symptoms and brain structure in non-infected HCs. This suggests that the brain–behaviour relationships observed in long COVID are likely specific to its neural mechanisms, rather than reflecting general physiological variability.^12^ Importantly, these associations were observed despite the absence of significant group-level morphometric differences between long COVID participants and healthy controls, suggesting that neuropsychiatric burden in long COVID may be reflected in specific patterns of structural covariance. Notably, executive dysfunction, working memory impairment, anxiety and sleep disturbances consistently exhibited high canonical and cross-loadings across RCCA models, indicating that this symptom cluster plays a pivotal behavioural role in linking the severity of neuropsychiatric symptoms with patterns of structural brain covariance. Supporting this finding, our previous work showed that anxiety symptoms following COVID-19 were largely driven by a chain-mediated effect involving co-occurring fatigue, sleep disturbances and PTSD.^23^ This further suggests that psychiatric and cognitive manifestations in long COVID may share overlapping pathophysiological mechanisms and neural networks. Although cognitive differences were less pronounced than psychiatric symptoms across both time points in long COVID and HCs, this pattern aligns with prior reports showing either no significant cognitive impairment or only mild, domain-specific deficits after COVID-19, with executive function being particularly vulnerable.^43-45^ Nonetheless, persistent subjective cognitive complaints remain strikingly common. This discrepancy may reflect co-occurring psychological distress that amplifies the subjective awareness of cognitive failures. Alternatively, some patients may exhibit anosognosia—a reduced awareness of objective cognitive deficits despite disrupted functional connectivity—further contributing to the notable heterogeneity in cognitive outcomes.^8,9,46^

Grey matter volume, which integrates cortical thickness and surface area, has been associated with psychiatric symptoms such as fatigue, sleep disturbances and anxiety in COVID-19 patients—particularly in the frontal cortex, precentral gyrus and precuneus.^23,47^ In the present study, although these exploratory multivariate associations should be interpreted cautiously given their limited stability across cross-validation folds and sensitivity to sample partitioning, we observed a temporal shift in the relationship between GMV and symptom severity between 1 and three months post-infection. At 1 month, greater GMV was linked to more severe neuropsychiatric symptoms, whereas by 3 months, smaller GMV correlated with worse symptom severity. As a composite marker of structural integrity, GMV changes in the acute phase may reflect neuroinflammation or vascular oedema—pathological processes that transiently increase GMV as a compensatory response to early neural dysfunction.^48^ By 3 months, the reversal towards reduced GMV accompanying severe symptoms may indicate a transition from acute compensation to progressive structural compromise.^49,50^ Across both time points, regions within the prefrontal–limbic network—particularly the dorsolateral prefrontal cortex (DLPFC) and anterior cingulate cortex (ACC)—emerged as the primary spatial contributors to the association between GMV and behavioural symptoms. These regions have previously been linked to post-COVID fatigue and anxiety.^23^ Collectively, these findings suggest that the frontal–limbic network may represent a potential neural substrate underlying the multidimensional symptom profile of long COVID, although further validation in larger, independent cohorts will be necessary.^47,51^

While GMV captures dynamic directional shifts, CTh may provide complementary insight into more stable neural correlates of long COVID. As a neuroanatomical marker reflecting local neuronal density and synaptic pruning, CTh has also been linked to the efficiency of neural processing underlying domain-specific cognitive functions in both Alzheimer’s disease and the oldest-old population.^18,52^ Previous studies have reported reduced cortical thickness in the left posterior superior temporal gyrus, bilateral postcentral gyri and olfactory and limbic regions in long COVID, particularly among individuals assessed 6 months to 2 years post-infection, with these changes associated with cognitive impairment.^17,19^ Our findings, which identified these same regions as major contributors to the cognitive-affective symptom cluster as early as 1 month post-infection, suggest that structural covariance in these circuits may emerge early rather than representing a late-onset phenomenon. Moreover, these results highlight the potential value of early structural markers for identifying individuals at risk for persistent cognitive-affective deficits. By 3 months, regions with high cross-loadings shifted from the primary sensorimotor cortex towards higher-order cognitive and affective regulatory networks, with the frontoparietal circuitry emerging as the dominant region.^53-55^ Given that bilateral parietal regions, particularly the inferior and superior parietal lobules, are key targets for cognitive intervention^56-58^—and that adaptive working memory training can enhance local CTh and cognitive performance—early cognitive interventions targeting parietal regions such as the supramarginal and angular gyri, which remained core regions across both the acute and post-acute phases, may provide preliminary insights for future cognitive intervention strategies in the subset of individuals exhibiting cognitive-affective impairments similar to those identified in our study.

Building upon these multivariate findings, we identified a consistent spatial pattern—including the superior and middle frontal gyri, anterior cingulate cortex, cuneus, angular gyrus and supramarginal gyrus—that contributed most prominently to neuropsychiatric symptoms across both the acute (1 month) and post-acute (3 months) phases following infection. The persistent involvement of these regions aligns with the frontal, parietal and occipital structural patterns repeatedly reported in neuroimaging studies of long COVID.^59,60^ Within this map, the superior and middle frontal gyri serve as key nodes of the executive control network,^61,62^ whereas the ACC mediates emotional regulation and cognitive flexibility.^63^ Meanwhile, the cuneus, angular gyrus and supramarginal gyrus are involved in visuospatial processing, attentional control and inter-network information integration, respectively.^64,65^ Structural variations in these regions have been consistently associated with hallmark long COVID symptoms, such as ‘brain fog’ and sensory disturbances. Notably, during the subacute phase, lower GMV and CTh within critical regions—including the superior frontal gyrus and cuneus—showed longitudinal associations with greater executive dysfunction and fatigue 1 year later. This relationship underscores the potential relevance of early structural markers in identifying individuals at risk of developing persistent post-COVID neuropsychiatric symptoms.

Several limitations of this study warrant consideration. First, the multivariate associations identified through RCCA should be interpreted as exploratory; their limited stability across cross-validation folds suggests sensitivity to sampling variability and our relatively modest sample size. While the observed longitudinal strengthening of these associations is encouraging, further validation in larger, independent cohorts, as well as the use of complementary multivariate approaches are essential to confirm their robustness. In addition, the visualization of regional contributors relied on a percentile-based threshold (top 5% of cross-loadings), which is somewhat arbitrary and should be interpreted cautiously. Second, although our behavioural follow-up extended to 1 year, the structural neuroimaging was limited to 3 months; future research with extended imaging protocols is needed to track long-term brain trajectories. Third, this study focused on structural markers without incorporating functional neuroimaging or biological markers (e.g. inflammatory/immune indices), which could offer a more comprehensive mechanistic understanding. Fourth, longitudinal assessments were not performed for healthy controls due to the large-scale COVID-19 outbreak during data collection, which resulted in most controls losing their non-infected status. To mitigate this, we balanced groups on key demographic variables at baseline. Finally, the neuropsychiatric phenotypes examined in this study represent only a subset of the broad post-COVID spectrum. Given the marked clinical heterogeneity of long COVID, future studies should employ clustering analyses to identify potential neurobiological subtypes and better characterize this heterogeneity.

## Conclusion

Long COVID may exhibit a temporally evolving yet topographically stable pattern of structure–behaviour associations, where early alterations in regional brain morphology contribute to long-term neuropsychiatric and cognitive outcomes. The identified regions—particularly within frontoparietal and limbic circuits—may serve as potential neuroimaging biomarkers for predicting long-term outcomes, providing a mechanistic a basis for the early identification of high-risk individuals and targeted intervention.

## Supplementary Material

fcag232_Supplementary_Data

## Data Availability

To get access to the data and comply with the terms of our research ethics committee approval, an application to the corresponding author will be required, specifying the geographical extent of sharing. The regularized canonical correlation analysis (RCCA) was performed using the publicly available CCA/PLS toolkit developed by the Machine Learning & Neuroimaging Laboratory, University College London (https://github.com/anaston/cca_pls_toolkit), which is openly accessible.

## Clinical trials registration

Brief title: Cohort Follow-up of Epidemic and Neuroimaging for Patients During the 1st Wave of the COVID-19 in China **gov ID:** NCT05745805; registered 23 February 2023.
