# Supplementary material for: Dynamic multivariate patterns of brain structure–neuropsychiatric symptom associations in long COVID
Source: Brain Commun. 2026 Jun 18;8(4):fcag232. doi: 10.1093/braincomms/fcag232 (PMC13343389; doi:10.1093/braincomms/fcag232)

Supplementary Table 1. Comparison of brain morphometric measures between long COVID participants (at one month, three months, and one year post-infection) and healthy controls (baseline).

Characteristic	HCs (n=68)	1 month (n=144)	*P* Value	3 months (n=144)	*P* Value	1 year (n=54)	*P* Value
Morphological brain measures							
eTIV	1490734.606 ±149832.5229	1513264.829 ±150326.6573	0.353	1509478.983 ±150326.6573	0.397	NA	NA
Lh_Mean thickness	2.42±0.10	2.43±0.10	0.572	2.44±0.09	0.226	NA	NA
Rh_Mean thickness	2.41±0.10	2.43±0.09	0.571	2.44±0.09	0.198	NA	NA

eTIV, estimated Total Intracranial Volume. Independent-sample t-tests were conducted to compare long COVID participants at each follow-up (1 month, 3 months, and 1 year post-infection) with baseline healthy controls. Reported *P*-values correspond to these comparisons.

Statistically significant results are highlighted in bold. *$P < 0.05$ (uncorrected); ** FDR-corrected $P < 0.05$.

Supplementary Table 2. MRI 3T scanners and parameters at each center.

Center	1	2	3	4	5	6	7	8	9
Scanner	GE Discovery MR750w	GE Discovery MR750w	Siemens Skyra	Siemens Skyra	GE SIGNA Premier	GE SIGNA Pioneer	Siemens Spectra	GE SIGNA Architect	GE SIGNA Architect
Structural T1-Weighted Images protocol									
TR (ms)	8.5	8.2	2.3	2.3	2.5	7.3	1.9	2.5	2.5
TI (ms)	600	450	900	900	1000	125	900	1000	1000
TE (ms)	3.2	3.2	2.3	2.3	3.0	3.1	2.4	3.1	3.1
Flip angle (^{o})	8	15	8	8	8	9	9	8	8
Voxel size (mm)	1×1×1	1×1×1	1×1×1	1×1×1	1×1×1	1×1×1	1×1×1	1×1×1	1×1×1
Matrix	256×256	256×256	256×256	256×256	256×256	256×256	256×256	256×256	256×256

TR, repetition time; TI, inversion time; TE, echo time.

Supplementary Table 3. Cross-validated canonical correlations between cortical thickness and neuropsychiatric symptoms at one and three month post-infection in individuals with long COVID.

Timepoint	Split	Canonical correlation (r)	*P*-value (permutation)
one month	1	0.313	0.043
	2	0.492	0.050
	3	0.310	0.054
	4	0.428	0.018
	5	0.523	0.002
	Mean (SD)	0.413 ± 0.099	NA

three months	1	0.353	0.032
	2	0.116	0.260
	3	0.338	0.043
	4	0.057	0.363
	5	0.605	0.001
	Mean (SD)	0.294 ± 0.218	NA

Supplementary Table 4. Cross-validated canonical correlations between gray matter volume and neuropsychiatric symptoms at one and three month post-infection in individuals with long COVID.

Timepoint	Split	Canonical correlation (r)	*P*-value (permutation)
one month	1	0.124	0.257
	2	-0.171	0.823
	3	0.060	0.398
	4	0.380	0.028
	5	0.302	0.052
	Mean (SD)	0.139 ± 0.216	NA
three months	1	0.210	0.160
	2	0.466	0.009
	3	0.330	0.052
	4	0.225	0.120
	5	0.350	0.041
	Mean (SD)	0.316 ± 0.104	NA

Supplementary Table 5. Regional canonical loadings and cross-loadings for cortical thickness (CTh) at one and three months post-infection under 5% and 10% thresholds.

Timepoint	Region	Canonical loadings (r)	*P* value	Cross-loading (r)	*P* value
1 month	left postcentral gyrus	-0.562	< 0.001	-0.364	< 0.001
	right lateral superior temporal gyrus	-0.544	< 0.001	-0.357	< 0.001
	right angular gyrus	-0.572	< 0.001	-0.354	< 0.001
	right postcentral gyrus	-0.464	< 0.001	-0.345	< 0.001
	left precentral gyrus	-0.572	< 0.001	-0.322	< 0.001
	right supramarginal gyrus	-0.542	< 0.001	-0.303	< 0.001

	right planum temporale	-0.503	< 0.001	-0.296	< 0.001
	left supramarginal gyrus	-0.537	< 0.001	-0.286	< 0.001
	left inferior frontal gyrus	-0.518	< 0.001	-0.278	< 0.001
	left middle temporal gyrus	-0.446	< 0.001	-0.273	0.001
	left angular gyrus	-0.486	< 0.001	-0.268	0.001
	left Heschl's gyrus	-0.408	< 0.001	-0.266	0.002
	left inferior frontal gyrus	-0.428	< 0.001	-0.256	0.002
	left paracentral lobule	-0.392	< 0.001	-0.250	0.002
	right inferior circular sulcus of the insula	-0.306	< 0.001	-0.242	0.003
3 months	left supramarginal gyrus	-0.775	< 0.001	-0.415	< 0.001
	right superior temporal sulcus	-0.767	< 0.001	-0.403	< 0.001
	right angular gyrus	-0.562	< 0.001	-0.381	< 0.001
	left angular gyrus	-0.736	< 0.001	-0.375	< 0.001
	right middle frontal gyrus	-0.714	< 0.001	-0.358	< 0.001
	left inferior frontal gyrus, pars operculari	-0.715	< 0.001	-0.353	< 0.001
	right superior occipital gyrus	-0.593	< 0.001	-0.346	< 0.001
	left postcentral sulcus	-0.683	< 0.001	-0.346	< 0.001
	left subcentral gyrus	-0.778	< 0.001	-0.342	< 0.001
	left intraparietal sulcus and transverse parietal sulci	-0.789	< 0.001	-0.337	< 0.001
	left superior temporal sulcus	-0.789	< 0.001	-0.334	< 0.001
	left Sylvian fissure	-0.551	< 0.001	-0.332	< 0.001
	right Sylvian fissure	-0.676	< 0.001	-0.329	< 0.001
	right superior frontal sulcus	-0.770	< 0.001	-0.325	< 0.001
	right intraparietal sulcus and transverse parietal sulci	-0.642	< 0.001	-0.322	< 0.001

Supplementary Table 6. Regional canonical loadings and cross-loadings for gray matter volume (GMV) at one and three months post-infection under 5% and 10% thresholds.

Timepoint	Region	Canonical loadings (r)	*P* value	Cross-loading (r)	*P* value
1 month	left inferior frontal gyrus, opercular part	-0.604	< 0.001	-0.447	< 0.001
	right middle frontal gyrus	-0.776	< 0.001	-0.441	< 0.001
	left superior frontal gyrus	-0.812	< 0.001	-0.403	< 0.001
	left transverse temporal gyrus	-0.585	< 0.001	-0.390	< 0.001
	right superior frontal gyrus	-0.825	< 0.001	-0.390	< 0.001
	left anterior cingulate gyrus and sulcus	-0.772	< 0.001	-0.388	< 0.001
	right cuneus	-0.644	< 0.001	-0.387	< 0.001
	right anterior cingulate gyrus and sulcus	-0.847	< 0.001	-0.386	< 0.001
	left orbital gyri	-0.774	< 0.001	-0.385	< 0.001
	right long insular gyrus and central sulcus of the insula	-0.672	< 0.001	-0.379	< 0.001
	left inferior frontal sulcus	-0.635	< 0.001	-0.377	< 0.001
	right middle–posterior cingulate gyrus and sulcus	-0.759	< 0.001	-0.371	< 0.001
	right subcallosal gyrus	-0.652	< 0.001	-0.366	< 0.001
	left long insular gyrus and central sulcus of the insula	-0.697	< 0.001	-0.364	< 0.001
	right orbital gyri	-0.797	< 0.001	-0.363	< 0.001
3 months	right superior frontal gyrus	0.730	< 0.001	0.392	< 0.001
	right cuneus	0.659	< 0.001	0.376	< 0.001
	left superior frontal gyrus	0.726	< 0.001	0.366	< 0.001
	right anterior cingulate gyrus and sulcus	0.726	< 0.001	0.356	< 0.001

	right orbital gyrus	0.706	< 0.001	0.345	< 0.001
	left anterior circular sulcus of the insula	0.495	< 0.001	0.342	< 0.001
	left middle frontal gyrus	0.676	< 0.001	0.331	< 0.001
	right lateral superior temporal gyrus	0.677	< 0.001	0.321	< 0.001
	left lateral superior temporal gyrus	0.611	< 0.001	0.319	< 0.001
	right dorsal posterior cingulate gyrus	0.580	< 0.001	0.319	< 0.001
	right middle–posterior cingulate gyrus and sulcus	0.586	< 0.001	0.318	< 0.001
	right superior occipital gyrus	0.599	< 0.001	0.317	< 0.001
	right transverse frontopolar gyri and sulci	0.567	< 0.001	0.314	< 0.001
	left orbital gyri	0.613	< 0.001	0.310	< 0.001
	left subcentral gyrus and sulcus	0.649	< 0.001	0.309	< 0.001

Supplementary Table 7. The detailed canonical cross-loadings and corresponding *P* values for each neuropsychiatric symptom

Neuropsychiatric symptoms	1 month		3 months	
	GMV (Panel A)	CTh (Panel B)	GMV (Panel C)	CTh (Panel D)
	Cross-loadings (*P*-value)	Cross-loadings (*P*-value)	Cross-loadings (*P*-value)	Cross-loadings (*P*-value)
PTSD	0.192 (0.021)	0.391 (<0.001)	-0.178 (0.033)	0.026 (0.753)
Fatigue	0.111 (0.184)	0.154 (0.065)	-0.240 (0.004)	0.088 (0.292)
Anxiety	0.269 (0.001)	0.320 (<0.001)	-0.220 (0.008)	0.252 (0.002)
Depression	0.128 (0.126)	0.296 (<0.001)	-0.236 (0.004)	0.121 (0.149)
Sleep disorder	0.210 (0.011)	0.301 (<0.001)	-0.306 (<0.001)	0.178 (0.033)
Forward-Digit Span Test	-0.201 (0.016)	-0.199 (0.017)	0.320 (<0.001)	-0.099 (0.235)

Backward-Digit Span Test	-0.270 (0.001)	-0.196 (0.019)	0.411 (<0.001)	-0.184 (0.028)
Immediate recall	-0.136 (0.106)	-0.426 (<0.001)	0.162 (0.053)	0.072 (0.389)
Short-term delayed recall	-0.081 (0.337)	-0.431 (<0.001)	0.093 (0.267)	0.067 (0.427)
Long-term delayed recall	-0.118 (0.160)	-0.503 (<0.001)	0.093 (0.267)	0.088 (0.296)
Recognition	0.055 (0.516)	0.238 (0.004)	-0.177 (0.034)	0.215 (0.010)
Trail Making Test-A	0.178 (0.033)	0.413 (<0.001)	-0.375 (<0.001)	0.245 (0.002)
Trail Making Test-B	0.325 (<0.001)	0.240 (0.004)	-0.453 (<0.001)	0.260 (0.001)

GMV: gray matter volume; CTh: cortical thickness; PTSD: post-traumatic stress disorder.

Supplementary Table 8. The detailed canonical loadings and corresponding *P* values for each neuropsychiatric symptom

Neuropsychiatric symptoms	1 month		3 months	
	GMV (Panel A)	CTh (Panel B)	GMV (Panel C)	CTh (Panel D)
	Canonical loadings (*P*-value)	Canonical loadings (*P*-value)	Canonical loadings (*P*-value)	Canonical loadings (*P*-value)
PTSD	0.432 (<0.001)	0.485 (<0.001)	-0.306 (<0.001)	0.263 (0.001)
Fatigue	0.403 (<0.001)	0.264 (0.001)	-0.443 (<0.001)	0.426 (<0.001)
Anxiety	0.639 (<0.001)	0.498 (<0.001)	-0.505 (<0.001)	0.626 (<0.001)
Depression	0.448 (<0.001)	0.453 (<0.001)	-0.443 (<0.001)	0.488 (<0.001)
Sleep disorder	0.446 (<0.001)	0.426 (<0.001)	-0.454 (<0.001)	0.516 (<0.001)
Forward-Digit Span Test	-0.474 (<0.001)	-0.296 (0.017)	0.513 (<0.001)	-0.259 (0.002)
Backward-Digit Span Test	-0.517 (<0.001)	-0.258 (0.002)	0.651 (<0.001)	-0.461 (<0.001)
Immediate recall	-0.360 (<0.001)	-0.563 (<0.001)	0.221 (0.008)	0.074 (0.376)
Short-term delayed recall	-0.307 (<0.001)	-0.647 (<0.001)	0.156 (0.061)	0.132 (0.113)

Long-term delayed recall	-0.294 (<0.001)	-0.636 (<0.001)	0.187 (0.025)	0.095 (0.257)
Recognition	0.081 (0.332)	0.205 (0.014)	-0.246 (0.003)	0.354 (<0.001)
Trail Making Test-A	0.340 (<0.001)	0.520 (<0.001)	-0.557 (<0.001)	0.470 (<0.001)
Trail Making Test-B	0.580 (<0.001)	0.335 (<0.001)	-0.660 (<0.001)	0.535 (<0.001)

GMV: gray matter volume; CTh: cortical thickness; PTSD: post-traumatic stress disorder.

Supplementary Figure 1. Frequency chart of symptoms among participants with long COVID (n=144).

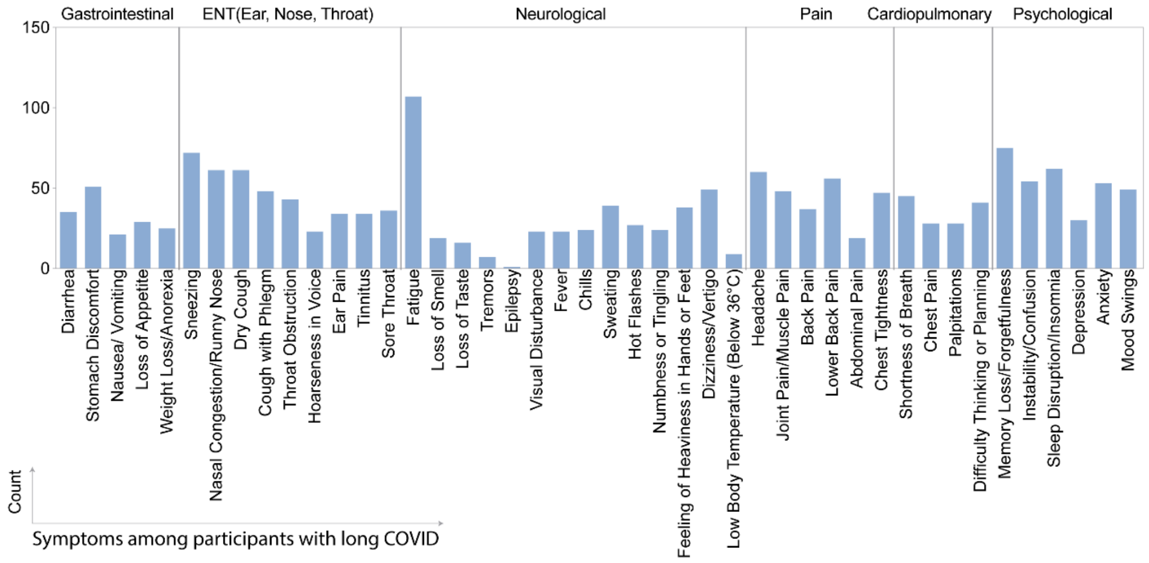

Supplementary Figure 2. Canonical loadings of neuropsychiatric symptoms in individuals with long COVID (n = 144).

Bar plots illustrate the canonical loadings of individual neuropsychiatric symptoms on the primary brain canonical variate. Panels (A) and (C) show results for gray matter volume (GMV) at one month and three months post-infection, respectively, whereas panels (B) and (D) show results for cortical thickness (CTh) at the corresponding time points.

Bar length reflects the magnitude of the canonical -loading for each symptom, and color intensity indicates the corresponding permutation-based *P* value, with darker colors representing stronger statistical significance. Statistically significant loadings ($P < 0.05$) are highlighted. Canonical loadings were derived from the regularized canonical correlation analysis (RCCA) model, and statistical significance was assessed using permutation testing (1000 iterations). The detailed canonical loadings and corresponding *P* values for each neuropsychiatric symptom, are provided in Supplementary Table 8.

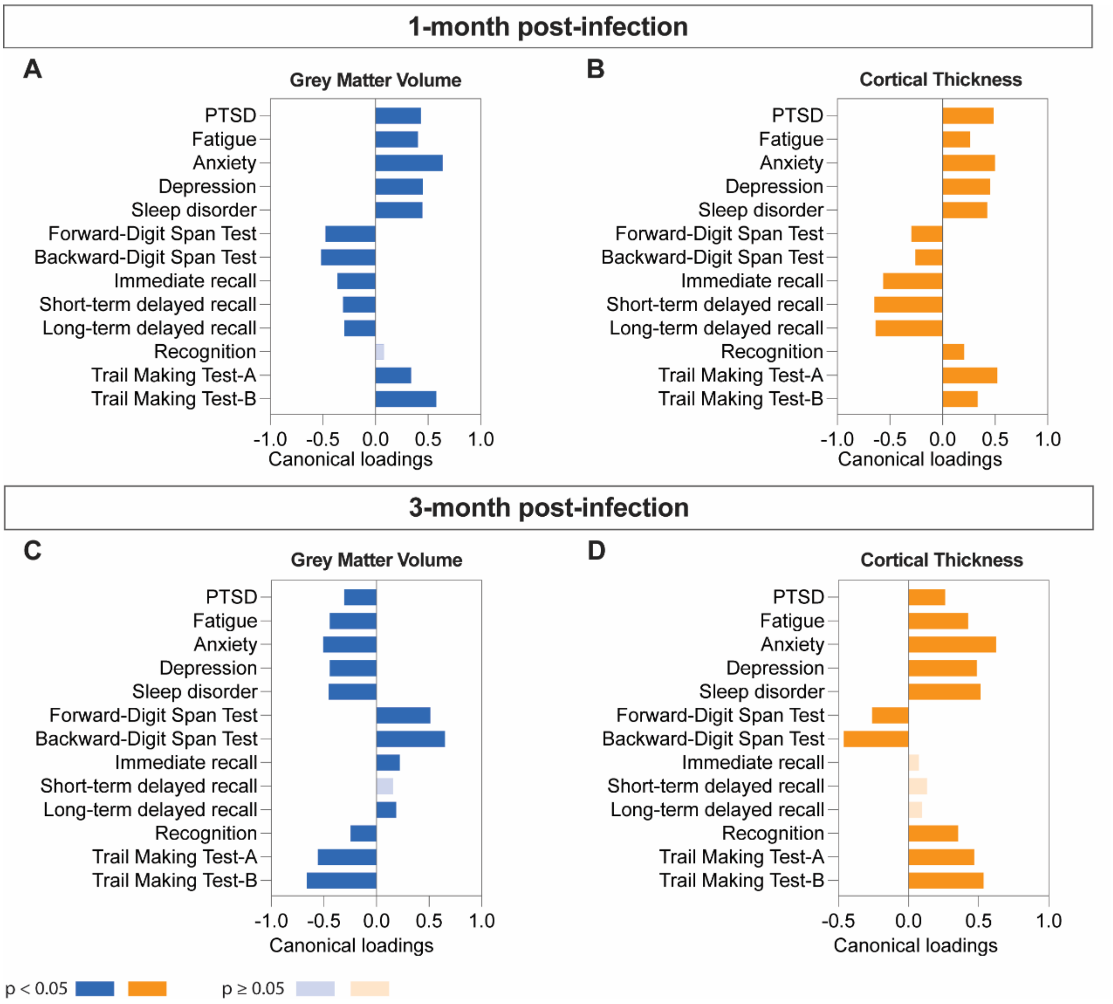

Supplementary Figure 3. Canonical loadings of brain regions in individuals with long COVID (n = 144).

Brain maps display regional canonical loadings associated with the primary brain–behavior canonical variate. Panels (A) and (C) show gray matter volume (GMV)–behavior associations at one and three months post-infection, respectively. Panels (B) and (D) show cortical thickness (CTh)–behavior associations at the corresponding time points.

Color intensity reflects the magnitude and direction of the canonical loadings (warm colors indicate positive associations; cool colors indicate negative associations).

Statistical significance of the canonical correlations was assessed using permutation testing (1000 permutations). Results are shown for the optimal model derived from the cross-validation framework. The detailed regional canonical loadings, thresholded at the top 5% and 10% of the distribution, along with their corresponding *P* values, are provided in Supplementary Tables 5 and 6.

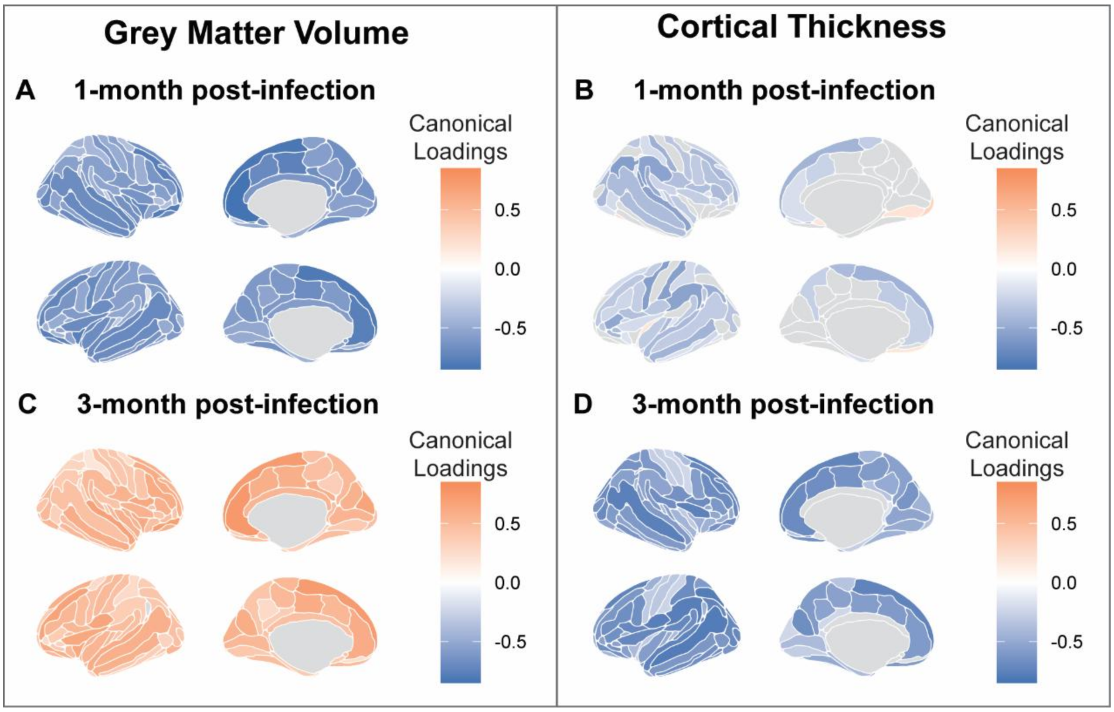

Supplement: fcag232_Supplementary_Data [file fcag232_supplementary_data.pdf]
